# Biological Characterization, Mechanistic Investigation and Structure‐Activity Relationships of Chemically Stable TLR2 Antagonists

**DOI:** 10.1002/cmdc.202000060

**Published:** 2020-06-03

**Authors:** Marcel Bermudez, Maria Grabowski, Manuela S. Murgueitio, Markus Tiemann, Péter Varga, Thomas Rudolf, Gerhard Wolber, Günther Weindl, Jörg Rademann

**Affiliations:** ^1^ Institute of Pharmacy (Pharmaceutical and Medicinal Chemistry) Freie Universität Berlin Königin-Luise-Strasse 2+4 14195 Berlin Germany; ^2^ Institute of Pharmacy (Pharmacology and Toxicology) Freie Universität Berlin Königin-Luise-Strasse 2+4 14195 Berlin Germany; ^3^ Section Pharmacology and Toxicology Pharmaceutical Institute Universität Bonn Gerhard-Domagk-Strasse 3 53121 Bonn Germany

**Keywords:** chemical synthesis, inflammation, molecular modeling, structure-based design, TLR selectivity, Toll-like receptors

## Abstract

Toll‐like receptors (TLRs) build the first barrier in the innate immune response and therefore represent promising targets for the modulation of inflammatory processes. Recently, the pyrogallol‐containing TLR2 antagonists CU‐CPT22 and MMG‐11 were reported; however, their 1,2,3‐triphenol motif renders them highly susceptible to oxidation and excludes them from use in extended experiments under aerobic conditions. Therefore, we have developed a set of novel TLR2 antagonists (**1**–**9**) based on the systematic variation of substructures, linker elements, and the hydrogen‐bonding pattern of the pyrogallol precursors by using chemically robust building blocks. The novel series of chemically stable and synthetically accessible TLR2 antagonists (**1**–**9**) was pharmacologically characterized, and the potential binding modes of the active compounds were evaluated structurally. Our results provide new insights into structure‐activity relationships and allow rationalization of structural binding characteristics. Moreover, they support the hypothesis that this class of TLR ligands bind solely to TLR2 and do not directly interact with TLR1 or TLR6 of the functional heterodimer. The most active compound from this series (**6**), is chemically stable, nontoxic, TLR2‐selective, and shows a similar activity with regard to the pyrogallol starting points, thus indicating the variability of the hydrogen bonding pattern.

## Graphical abstract

**Undegraded**: The 1,2,3‐triphenol motif of known TLR2 antagonists is highly susceptible to oxidation and excludes them from use in extended experiments under aerobic conditions. Herein, we report a rationally developed series of novel TLR modulators resulting in compound **6**, a novel, chemically stable, nontoxic, TLR2‐selective antagonist.

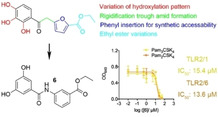

## Introduction

**Figure 1:**
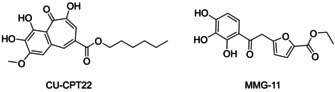
Previously discovered competitive TLR2 antagonists CU‐CPT22 and MMG‐11 both containing the pyrogallol scaffold.^7^, 10a

The first barrier in the innate immune response is formed by the family of structurally conserved Toll‐like receptors (TLRs).^1^ In humans ten functional subtypes (TLR1–TLR10) have been identified. TLRs recognize intruding pathogens or endogenous danger signals released after cell damage or cell death and activate the innate immune response against them. This happens through the specific binding of pathogen‐associated molecular patterns (PAMPs) and danger‐associated molecular patterns (DAMPs), respectively.^2^ TLR2 forms heterodimers with TLR1 and TLR6 and specifically recognizes several components of the cell wall of gram positive bacteria like di‐ and tri‐acylated lipoproteins, lipoteichoic acids or lipomannans. After ligand binding, the preformed dimer undergoes conformational changes that trigger an intracellular signaling cascade that leads to the activation of NF‐κB and the secretion of pro‐inflammatory cytokines like tumor necrosis factor (TNF) and interleukin (IL)‐8.^3^ Under certain circumstances this response is excessive and leads to severe conditions like sepsis, rheumatoid arthritis, autoimmune diabetes, asthma and certain types of allergies.^1^, ^4^ The modulation of TLR2 function by small molecules has been postulated as a promising strategy to treat these conditions. To date only few compounds that modulate TLR2 activity have been identified and pharmacologically characterized. In 2010, four small organic molecules with agonistic activity on the receptor were discovered by high‐throughput screening by Guan et al.^5^ One of them was later chemically optimized.^6^ In 2012 the first competitive antagonist CU‐CPT22 was discovered by Yin et al. (Figure 1, left).^7^ Virtual screening has successfully been applied to discover agonists and antagonists for TLR2,^8^ but also for other TLR subtypes.^9^ In a previous study, we identified a potent, competitive and selective TLR2 antagonist MMG‐11;^10^ however, it still contained the pyrogallol fragment (Figure 1, right). As the pyrogallol scaffold is notorious for its drawbacks including low chemical stability and poor synthetic accessibility, the modification of this scaffold to one that is less prone to oxidation is essential for further optimization steps.

In this work, we explored the chemical space around the pyrogallol‐containing antagonists, MMG‐11 and CU‐CPT22, to enhance synthetic accessibility and chemical stability, and get insights into the structure‐activity‐relationships (SARs) of TLR2 antagonists. We performed synthetic modifications and analogue searches. The synthesized small molecules and selected analogues were biologically tested for their ability to inhibit TLR2 signaling. This leads to several novel TLR2 antagonists, a better understanding of their SAR and provides a way to rationalize binding modes of TLR2 antagonists.

## Results and Discussion

### Compound selection, design and synthesis

**Scheme 1:**
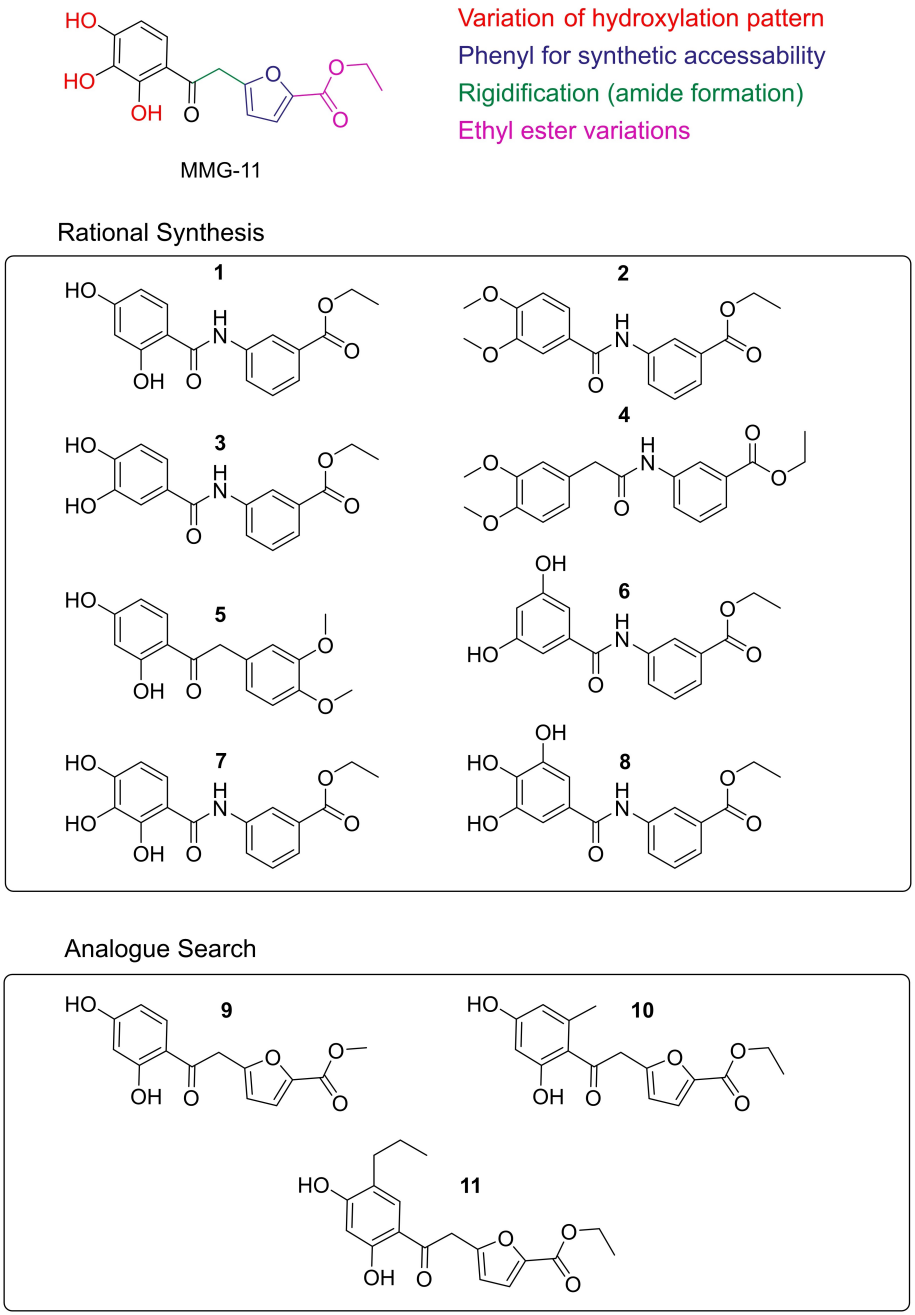
Synthesized and selected compounds. The starting point MMG‐11 is depicted on the top with the different variations highlighted in color. Compounds **1** to **8** were rationally designed and synthesized and are shown on the left side. The compounds selected by analogue search (**9** to **11**) are shown on the right side.

**Figure 2:**
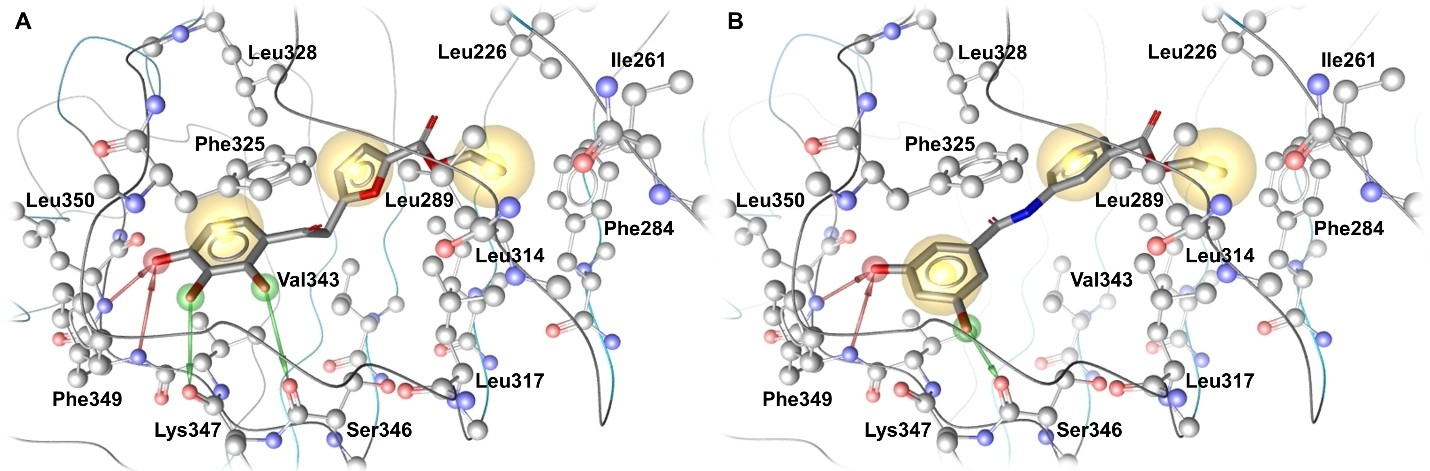
Predicted binding pose for MMG‐11 and **6**. The TLR2 antagonists A) MMG‐11 and B) **6** bound in the TLR2 ligand binding site are shown. Protein residues are depicted in ball and stick mode, the compound as sticks. Protein‐ligand interactions are color‐ and shape‐coded (yellow sphere – hydrophobic contact area, green arrow – H‐Bond donor, red arrow – H‐Bond acceptor).

**Scheme 2:**
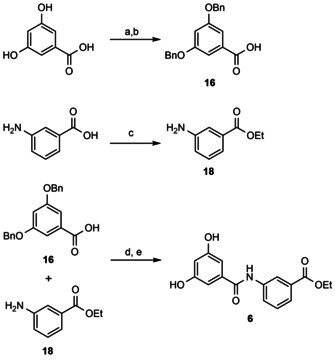
Synthesis of inhibitor **6**. a) BnBr, K_2_CO_3_, acetone, reflux, 8 h; b) NaOH, MeOH, H_2_O, reflux, 8 h, quant. over 2 steps. c) SOCl_2_, EtOH, reflux, 4 h, 92 %. d) HATU, DIPEA, CH_2_Cl_2_, 30 °C, 8 h, 36 %; e) H_2_, Pd/C, CH_2_Cl_2_, MeOH, 86 %. HATU=*O*‐(7‐azabenzotriazol‐1‐yl)‐*N,N,N’,N’*‐tetramethyluronium hexafluorphosphate

A selection of the compounds that resulted from both chemical synthesis and similarity‐based analogue search is shown in Scheme 1. The design was guided by binding mode investigations of MMG‐11 in complex with TLR2 regarding spatial requirements of the binding site and potential receptor‐ligand interactions. Especially, we intended to modify the polyphenolic core structure, with the aim of avoiding the most easily oxidized 1,2‐diphenols and 1,2,3‐triphenols or the phenoxy ethers derived from them. Since the three hydroxy groups of the pyrogallol scaffold are involved in hydrogen bonding with the receptor (Figure 2A),10a we had to systematically evaluate these interactions. Therefore, we reduced the number of hydroxy groups capable to function as both hydrogen bond donors and acceptors (**1**, **3**, **5** and **6**) and varied the substitution pattern. For a systematic control, two compounds still comprising the 1,2,3‐trihydroxy motif, **7** and **8**, were included in the study. Furthermore, we introduced methoxy groups, which can only serve as hydrogen bond acceptors (**2** and **4**). Considering the flexibility of the lead structure, we introduced an amide moiety to rigidify the molecules (**1**–**4** and **6**). In order to enhance synthetic accessibility and the chemical stability, we exchanged the furan moiety by a phenyl ring in all synthesized compounds. This resulted in a set of eight synthesis‐derived compounds (**1**–**8**, Schemes 1 and 2 and the Supporting Information).

In a complementary approach we searched for structural analogues in the databases which were used for the discovery of MMG‐11 by virtual screening.10a MMG‐11 was used as the query structure and the databases were searched for similar commercially available molecules with a Tanimoto coefficient higher than 0.8. We found three closely related compounds in the Enamine database (Enamine Ltd, Kiev, Ukraine) which were ordered for biological evaluation (**9**–**11**). Molecular weight and purity (>95 %) were confirmed by LC–MS.

The resulting set of eleven derivatives obtained by synthesis and analogue search, provides the possibility to conceive the SAR of TLR2 antagonists, in particular for the rationalization of the hydrogen bond pattern of polyphenolic ligands.

Compounds **1**, **3**, and **6** were synthesized starting from the corresponding 2,4‐, 3,4‐, or 3,5‐dihydroxy‐benzoic acids as exemplified for compound **6** in Scheme 2. First, both the phenolic hydroxy groups and the carboxylic acid residues were protected in one step as *O*‐benzyl‐ethers and esters, respectively, using benzyl bromide with iodide addition and furnishing the tri‐*O*‐benzyl‐protected intermediates **12**–**14**.

The tri‐O‐methyl‐protected 3,4‐dihydroxy benzoic acid **15** needed for the synthesis of compound **2** was prepared by an analogous protocol using methyl iodide for alkylation. Saponification of the esters **12**–**15** afforded the free carboxylic acids **16**–**19** in very good yields (95 % quantitatively). Next, the prepared carboxylic acids **16**–**19** or commercially available 3,4‐dimethoxy‐phenylacetic acid were activated using *O*‐(7‐azabenzotriazol‐1‐yl)‐*N,N,N′,N′*‐tetramethyluronium hexafluorophosphate (HATU) in the presence of ethyl 3‐aminobenzoate **20** yielding the protected benzamides **2**, **4**, and **21**–**23** as the desired condensation products. Here the yields were moderate, presumably due to the reduced nucleophilicity of the aromatic amine in **20**. Hydrogenolysis with palladium on charcoal removed the benzyl ether groups and furnished the unprotected benzamides **1**, **3**, and **6** in very good yields, for example, 86 % for compound **6**.

Aromatic ketone **5**, in which the amide linker between two benzene rings is replaced by a ketomethylene unit, was obtained via the direct C‐acylation of resorcinol (1,3‐diphenol) with 3,4‐dimethoxy‐phenyl acetic acid using boron trifluoride diethyl etherate as the activating Lewis acid in 19 % yield. Compounds **1**–**6** were isolated with >95 % purity by column chromatography. Synthesis of the trihydroxy‐derivatives, ethyl 3‐(2,3,4‐trihydroxy‐benzamido)benzoate **7** and ethyl 3‐(3,4,5‐trihydroxy‐benzamido)benzoate **8** was attempted following the same strategy as in Scheme 2. While the preparation of the tri‐O‐benzyl‐protected precursors of **7** and **8** proceeded smoothly, debenzylation of the protected intermediates led to the instantaneous decomposition of these products due to oxidation. **7** and **8** therefore could not be isolated and tested biologically.

### Biological validation

**Figure 3:**
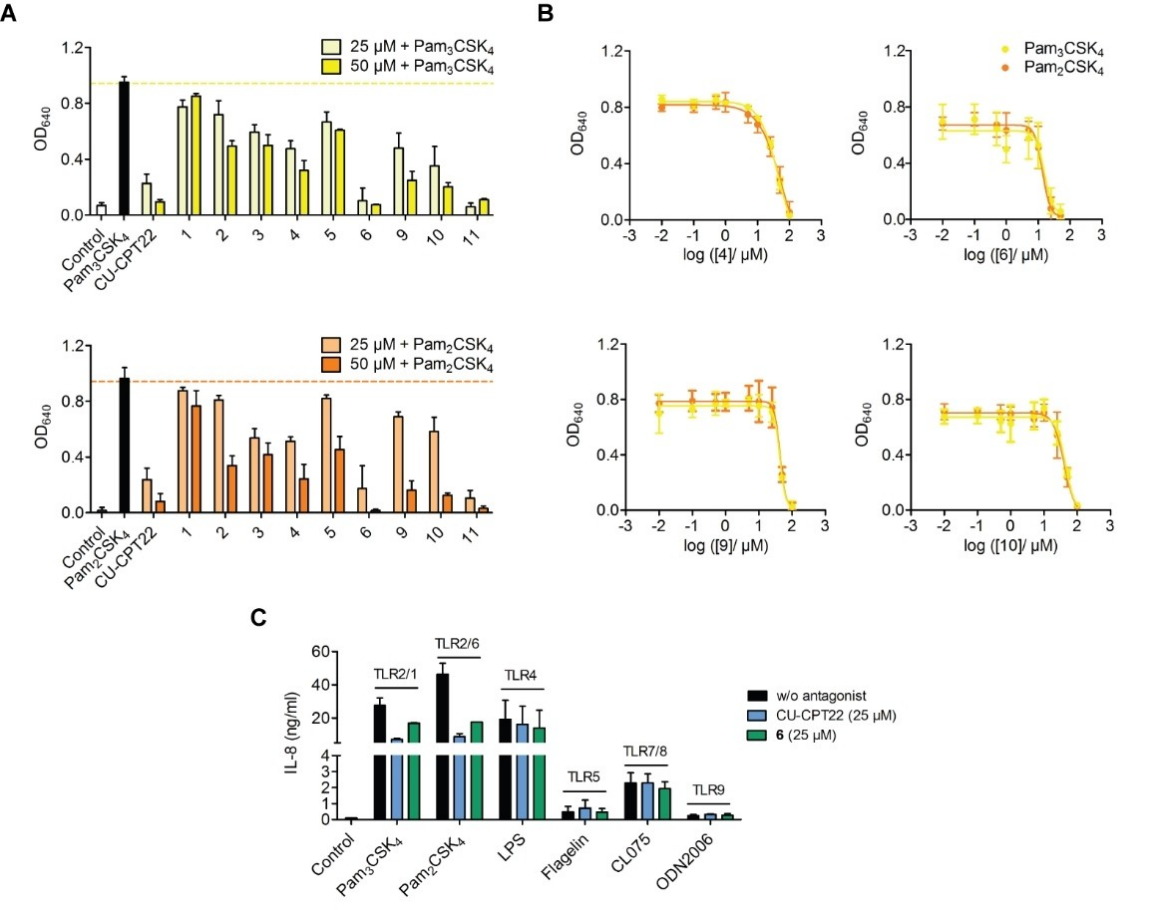
Inhibition of TLR2‐dependent NF‐κB activation and IL‐8 secretion. A) HEK‐Blue hTLR2 cells were pre‐incubated with CU‐CPT22 or compounds **1**–**6** and **9**–**11** for 1 h and then stimulated additionally with TLR2/1 agonist Pam_3_CSK_4_ (10 ng/mL) or TLR2/6 agonist Pam_2_CSK_4_ (1 ng/mL) for 24 h. B) HEK‐Blue hTLR2 cells were pre‐incubated with increasing concentrations of **4**, **6**, **9**, **10** and then stimulated additionally with Pam_3_CSK_4_ or Pam_2_CSK_4_ for 24 h. Supernatants were tested for TLR2‐mediated NF‐κB/AP‐1 activity by quantification of SEAP release (OD_640_). C) THP‐1 macrophages were incubated with CU‐CPT22 (25 μM) or compound **6** for 1 h and afterwards stimulated with Pam_3_CSK_4_ (10 ng/mL), Pam_2_CSK_4_ (1ng/mL), LPS (10 ng/mL), flagellin (1 μg/mL), CL075 (8 μM) or ODN2006 (5 μM) for 24 h. Supernatants were analyzed for IL‐8 secretion by ELISA. Mean+SD or ±SD (*n*=3).

To explore the activity of the synthesized and selected compounds **1** to **6** and **9** to **11** on the TLR2 activity, reporter cells overexpressing hTLR2 were used. CU‐CPT22 was used as the reference TLR2‐antagonist.^7^, 10a, 11 Except for **1**, all selected compounds decreased TLR2‐mediated NF‐κB activation in a primary screen (Figure 3A). Additionally, **4**, **6**, **9** and **10** reduced Pam_3_CSK_4_‐induced TLR2/1 responses to less than 40 % at 50 μM and **2**–**6**, **9** and **10** diminished Pam_2_CSK_4_‐induced TLR2/6 responses to less than 50 % at 50 μM. Compound **6** and **11** abrogated the TLR2 response of both heterodimers almost completely and therefore showed a similar TLR2 inhibition as CU‐CPT22 (Figure 3A). None of the tested compounds appeared to have any agonistic TLR2 activity (Figure S1A in the Supporting Information).

No cytotoxic effects were observed within the activity range except for **11** which reduced cell viability starting at 50 μM (Figure S1B). Hence, the apparent inhibition of TLR2‐mediated responses by **11** might be due its cytotoxicity. Consequently, compound **11** was excluded from further investigation. The remaining most active hits, **4**, **6**, **9** and **10**, were analyzed regarding their potency. For this purpose, concentration‐response curves were assessed and IC_50_ values were calculated (Figure 2B). All four compounds showed IC_50_ values in the μM range, thus confirming their antagonistic effect as MMG‐11 analogues (Table 1). Compound **6** turned out to have the lowest IC_50_ value of the identified TLR2 inhibitors with similar results for both heterodimers (TLR2/1: 15.4 μM; TLR2/6: 13.6 μM). This suggests binding of compound **6** to the TLR2 cavity but no interaction towards TLR1 or TLR6. While compound **6** effectively inhibits TLR2 signaling, we could show that it has no influence on other TLR subtypes (Figure 2C). While keeping the activity of compound **6** in a comparable range as for CU‐CPT22 and MMG‐11, we could replace the highly sensitive 1,2,3‐trihydroxyphenyl structure of pyrogallol in the starting compounds by the chemically stable 1,3‐dihydroxyphenyl substructure of resorcinol. This is essential for further optimization of TLR2 antagonists because **6** is robust, synthetically accessible and no longer susceptibility toward oxidation.

**Table 1:** IC_50_ values of the compounds **4**, **6**, **9** and **10** by TLR2/1 stimulation (Pam_3_CSK_4_, 10 ng/mL) and TLR2/6 stimulation (Pam_2_CSK_4_, 1 ng/mL) in HEK‐Blue hTLR2 cells (n=3).

Compound	TLR2/1 IC_50_ [μM]^[a]^	TLR2/6 IC_50_ [μM]^[a]^
**4**	44.9 (28.1–72.0)	61.9 (18.7–204.6)
**6**	15.4 (8.9–26.4)	13.6 (9.9–18.6)
**9**	42.4 (36.9–48.7)	42.9 (36.9–49.9)
**10**	44.0 (33.8–57.4)	40.7 (30.9–53.6)

[a] 95 % confidence interval shown in brackets.

### SAR of novel antagonists

In order to rationalize the bioactivity and elucidate the potential binding modes of the tested compounds docking studies were performed. As all characterized compounds both inhibited TLR2/1 and TLR2/6 signaling, without showing effects at other TLRs, we hypothesized that they would bind to the TLR2 monomer and share the same ligand binding pocket like its pyrogallol‐containing precursor, MMG‐11.10a This compound has been previously shown to be a competitive antagonist for the binding of Pam_3_CSK_4_ and Pam_2_CSK_4_, which further supports our proposed binding mode. However, an experimental confirmation of the binding mode is beyond the scope of this study. Extensive docking analyses were performed and plausible binding poses selected based on their similarity to the binding mode of MMG‐11 and the amount of performed interactions between the ligand and the TLR2 binding site. The resulting proposed binding mode for the most active compound **6** is shown in Figure 3B. Selected docking poses for the remaining compounds can be found in the Figures S2 to S5. **6** is embedded in the front part of the binding pocket with the dihydroxy‐benzamide moiety forming several H‐Bonds to the backbone atoms of TLR2. The benzene ring forms hydrophobic contacts to the Phe325 side chain.

Deeper in the pocket further hydrophobic contacts take place between the benzoate and Leu328, Val313 and Ile314 and the ethyl moiety and Phe284, Leu317, Leu285, Ile261, Leu266 and Ile314. H‐bond acceptor interactions are formed by the hydroxy group on position 5 and the backbone nitrogen atoms of Leu350 and Phe349, in addition to an H‐Bond donor interaction between the second hydroxy group in position 3 and backbone oxygen of Ser346. These H‐Bonds are also formed by MMG‐11 (Figure 2A) and have been shown to be important for antagonists binding to TLR2.8e This might explain the lower activity of the other dihydroxybenzamidobenzoates (**1** and **3**, Figure S2) and the dimethoxybenzamidobenzoates (**2** and **4**, Figure S3). The geometry of the 2,4‐dihydroxy‐benzamide **1** causes it to form H‐Bonds to Ser346 and Lys347 but not Leu350 and Phe349 leading to a weak activity. The 3,4‐dihydroxy‐benzamide **3** is more active than **1** as the mandatory H‐Bonds to Leu350 and Phe349 are formed, but less active than **6** as the stabilizing H‐Bond to Ser326 is missing. In the case of the dimethoxybenzamidobenzoates the methoxy groups are worse and bulkier acceptors than the hydroxy groups thus making the formation of the key H‐Bonds less favorable. For these compounds we hypothesize a flipped binding mode which allows the carbonyl oxygen of the ester to interact with the backbone of Phe349 and Leu350, without the formation of further stabilizing H‐Bonds towards Ser346 the resulting activity still is low. The dihydroxyphenyl moiety of compound **5** is surmised to form two H‐Bonds towards Phe349 and Leu350, however its scaffold puts the methoxy groups into proximity of hydrophobic residues, which is unfavorable for binding and leads to a reduced activity (Figure S4).

In addition to the synthesized compounds we tested three dihydroxy‐phenyl‐oxoethyl‐furan‐carboxylates (**9**, **10** and **11**) of which one **11** was cytotoxic and therefore omitted in further analysis. The other two compounds, **9** and **10** show a binding mode nearly identical to that of MMG‐11 (Figure S5). Surprisingly, their activity is much lower, which shows the importance of the third hydroxy group for the stabilization of ligands containing the furan carboxylate scaffold. Interestingly, this is not the case for the newly developed and rigidified dihydroxybenzamidobenzoates like lead structure **6**, which virtually retains the binding affinity despite of having only two remaining hydroxy groups.

## Conclusion

Taken together, this study reports a set of TLR2 antagonists derived by chemical synthesis and analogue search based on the previously reported pyrogallol‐containing TLR2 antagonists CU‐CPT22 and MMG‐11. The series of compounds (**1**–**11**) designed and tested here provides novel insights into the structure‐activity relationships of TLR2 antagonists and allows for rationalization of structural binding characteristics. The findings prove the possibility of varying the pyrogallol fragment, the hydrogen bonding pattern, the second aryl aryl fragment and the linker moiety connecting both fragments. These tolerated variations enabled the development of chemically stable drug candidates without the sensitive pyrogallol scaffold. The nontoxic and most active compound from this series, inhibitor **6**, shows a comparable activity toward TLR2 as its pyrogallol precursors without being sensitive toward oxidative degradation like its pyrogallol derivatives. Furthermore, the biological activity of this inhibitor supports our hypothesis that this group of TLR antagonists binds solely to TLR2 and does not directly interact with the TLR1 or TLR6 part of the functional heterodimer.

## Experimental Section

**Chemical synthesis**: Chemicals were purchased from Acros, Alfa Aesar, Fluka, Sigma‐Aldrich, and VWR and were used without further purification or distillation unless otherwise stated. All solvents that were used during the reactions were obtained from a column‐based solvent system (MBraun, MB‐SPS‐800). Air or moisture‐sensitive reactions were carried out under a positive pressure of dry nitrogen or argon in oven‐dried glassware. Solution phase reactions were monitored either by LC‐MS techniques or by thin‐layer chromatography (TLC) using Analtech silica gel plates (60 F254) and the spots were examined under UV light at 254 nm or stained with developing reagents. LC‐MS data were recorded on an Agilent 1100 series chromatography workstation (Agilent Technologies) equipped with a single quadruple mass spectrometer and electrospray ionization (ESI). Eluents A (0.1 % formic acid in Millipore water) and B (0.1 % formic acid in acetonitrile) were used in a linear gradient (5–99 % B in 5 min). Solvents were removed in rotary evaporators from Heidolph. The Biotage®Isolera^TM^ Spektra One was used for regular flash purifications with pre‐pack flash chromatography cartridges from Biotage® employing individual gradients derived from analytical runs or thin layer chromatography (TLC). Non‐HRMS measurements were recorded with an ESI single quad spectrometer (Agilent) coupled with an analytical HPLC system (Agilent 1100). HRMS measurements were conducted with an Agilent 6210 ESI‐TOF mass spectrometer. NMR spectra (^1^H and ^13^C NMR) were recorded on a Bruker AVANCE 300 MHz, 400 MHz and 500 MHz instrument and chemical shifts (*δ*) were measured in parts per million (ppm). The elemental analyzer VARIO EL was used for C, H, and N analysis. Details for compound synthesis and analytic data are given in the Supporting Information.

**General procedure for the preparation of perbenzylated benzoic acid esters 12**–**14**: Benzyl bromide (6 equiv), sodium iodide (6 equiv) and potassium carbonate (8 equiv) were added to a solution of the respective benzoic acid in acetone (40 mL mmol^−1^) at room temperature. The mixture was stirred under reflux for 16 h. Then it was cooled to room temperature and the solvent removed under reduced pressure. Water was added and the resulting mixture was extracted with EtOAc. The combined organic layers were dried over anhydrous Na_2_SO_4_ and filtered. EtOAc was evaporated under reduced pressure and the resulting crude product was purified by column chromatography over SiO_2_ (hexane/EtOAc as eluent) to afford the desired perbenzylated compounds.

**Benzyl 2,4‐bis‐(benzyloxy)benzoate (12)**: Compound **12** was synthesized according to the general procedure using 2,4‐dihydroxybenzoic acid (1.00 g, 6.5 mmol) and was obtained as a pale yellow solid (1.58 g, 3.72 mmol, 57 %). ^1^H NMR (400 MHz, CDCl_3_): *δ*=7.91 (d, *J*=8.7 Hz, 1H), 7.46–7.28 (m, 15H), 6.61 (s, 1H), 6.58 (d, *J*=8.7 Hz, 1H), 5.32 (s, 2H), 5.11 (s, 2H), 5.06 (s, 2H).

**Benzyl 3,4‐bis‐(benzyloxy)benzoate (13)**: Compound **13** was synthesized according to the general procedure using 3,4‐dihydroxybenzoic acid (5.00 g, 32.5 mmol) and was obtained as a pale yellow solid (7.80 g, 18.53 mmol, 57 %). ^1^H NMR (400 MHz, CDCl_3_): *δ*=7.71–7.65 (m, 2H), 7.49–7.31 (m, 15H), 6.93 (d, *J*=8.9 Hz, 1H), 5.32 (s, 2H), 5.22 (s, 2H), 5.20 (s, 2H).

**Benzyl 3,5‐bis‐(benzyloxy)benzoate (14)**: Compound **14** was synthesized according to the general procedure (without NaI) by using 3,5‐dihydroxybenzoic acid (0.77 g, 5 mmol) and was obtained as a pale yellow solid (0.41 g, 0.95 mmol, 19 %). ^1^H NMR (500 MHz, [D_6_]DMSO): *δ*=7.46–7.31 (m, 15H), 7.18 (s, 2H), 6.98 (s, 1H), 5.33 (s, 2H), 5.15 (s, 4H).

**Methyl 3,4‐dimethoxy‐benzoate (15)**: To a solution of 3,4‐dihydroxy‐benzoic acid (0.80 g, 5.2 mmol, 1 equiv) in DMF (30 mL) was added methyl iodide (1.50 mL, 25 mmol, 5 equiv) and potassium carbonate (3.59 g, 25 mmol, 5 equiv) at room temperature. The mixture was stirred at 50 °C for 16 h, cooled to room temperature and concentrated under reduced pressure. Water was added and the resulting mixture extracted with EtOAc (3×20 mL). The combined organic layers were dried over anhydrous Na_2_SO_4_ and filtered. The EtOAc was evaporated under reduced pressure and the resulting crude product purified by column chromatography on SiO_2_ (hexane/EtOAc as eluent) to afford **15** as a white solid (0.73 g, 3.74 mmol, 72 %). ^1^H NMR (400 MHz, CDCl_3_): *δ*=7.65 (d, *J*=8.4 Hz, 1H), 7.52 (s, 1H), 6.86 (d, *J*=8.4 Hz, 1H), 3.91 (s, 6H), 3.87 (s, 3H).

**Preparation of benzoic acids 16**–**18. General procedure**: To a suspension of the appropriate benzyl benzoates (**12**–**14**) in EtOH at room temperature was added an excess of 5 M NaOH solution and the resulting mixture stirred under reflux for 8 h. The reaction mixture was cooled to room temperature and acidified to pH 3 by dropwise addition of 12 M HCl. The resulting precipitate was filtered off, washed with water and dried under reduced pressure to afford the corresponding acids **16**–**18**.

**2,4‐Bis‐(benzyloxy)benzoic acid (16)**: Compound **16** was synthesized according to the general procedure, using **12** (1.01 g, 2.36 mmol). The resulting precipitate was recrystallized from MeOH/CH_2_Cl_2_ (50 : 1) and afforded **16** as a white solid (0.80 g, 1,89 mmol, 80 %). ^1^H NMR (400 MHz, [D_6_]DMSO): *δ*=12.27 (s, 1H), 7.72 (d, *J*=8.6 Hz, 1H), 7.54–7.27 (m, 10H), 6.82 (s, 1H), 6.67 (d, *J*=8.8 Hz, 1H), 5.20 (s, 2H), 5.16 (s, 2H).

**3,4‐Bis‐(benzyloxy)benzoic acid (17)**: Compound **17** was synthesized according to the general procedure, using **13** (2.30 g, 5.42 mmol) and was obtained as a white solid (1.70 g, 5.09 mmol, 94 %). ^1^H NMR (400 MHz, [D_6_]DMSO): *δ*=12.57 (s, 1H), 7.46 (s, 1H), 7.45 (d, *J*=7.4 Hz, 1H), 7.39–7.19 (m, 10H), 7.06 (d, *J*=8.8 Hz, 1H), 5.12 (s, 2H), 5.08 (s, 2H).

**3,5‐Bis‐(benzyloxy)benzoic acid (18)**: Compound **18** was synthesized according to the general procedure, using **14** (0.21 g, 0.50 mmol) and was obtained as a white solid comprising a mixture of acid and sodium salt (0.21 g, quant.). ^1^H NMR (500 MHz, [D_6_]DMSO): *δ*=13.04 (s, 1H) 7.46–7.32 (m, 10H), 7.16 (s, 2H), 6.93 (s, 1H), 5.14 (s, 4H).

**Preparation of 3,4‐dimethoxy‐benzoic acid (19)**: To a suspension of **15** (0.35 g, 1,79 mmol, 1 equiv) in H_2_O/MeOH (1 : 1 *v*/*v*, 30 mL) at room temperature was added LiOH monohydrate (0.23 g, 5.36 mmol, 3 equiv) and the resulting mixture stirred for 4 h. The reaction mixture was then brought to pH 3 using 1 M HCl and extracted with EtOAc. The combined organic layers were dried over anhydrous Na_2_SO_4_ and filtered. The EtOAc was evaporated under reduced pressure to afford **19** as a white solid (0.31 g, 1.70 mmol, 95 %). ^1^H NMR (400 MHz, [D_6_]DMSO): *δ*=12.67 (s, 1H), 7.56 (d, *J*=8.4 Hz, 1H), 7.44 (s, 1H), 7.03 (d, *J*=8.5 Hz, 1H), 3.82 (s, 3H), 3.79 (s, 3H).

**Preparation of ethyl 3‐amino‐benzoate (20)**: Compound **20** was synthesized according to literature.^1^ Brown oil, yield: 92 %,^1^H NMR (400 MHz, CDCl_3_): *δ*=7.41 (d, *J*=7.7 Hz, 1H), 7.34 (s, 1H), 7.19 (dd, *J*=7.8 Hz, 1H), 6.83 (d, *J*=8.0 Hz, 1H), 4.33 (q, *J*=7.1 Hz, 2H), 3.48 (s, 2H), 1.36 (t, *J*=7.1 Hz, 3H).

**General procedure for the preparation of benzamides 2**, **4**, **and 21**–**23**: To a mixture of the appropriate acid (1 equiv) and **20** (1 equiv) in CH_2_Cl_2_ (5 mL mmol^−1^) was added HATU (1.2 equiv) and DIPEA (3 equiv) at 0 °C and after which it was stirred at 30 °C for 8 h. The solvent was removed under reduced pressure, water was added and the resulting mixture was extracted with EtOAc. The combined organic layers were dried over anhydrous Na_2_SO_4_ and filtered. The EtOAc was evaporated under reduced pressure and the resulting crude product was purified by column chromatography over SiO_2_ (hexane/EtOAc as eluent) to afford the desired amides **2**, **4**, **21**–**23**.

**Ethyl 3‐(3,4‐dimethoxy‐benzamido)benzoate (2)**: Compound **2** was synthesized according to the general procedure by using **19** (0.05 g, 0.28 mmol) and was obtained as a yellow solid (0.63 g, 0.21 mmol, 76 %). ^1^H NMR (400 MHz, CDCl_3_): *δ*=8.19–8.00 (m, 3H), 7.80 (d, *J*=7.5 Hz, 1H), 7.49 (s, 1H), 7.47–7.37 (m, 2H), 6.87 (d, *J*=8.4 Hz, 1H), 4.35 (q, *J*=7.1 Hz, 2H), 3.92 (s, 6H), 1.37 (t, *J*=7.0 Hz, 3H). ^13^C NMR (101 MHz, CDCl_3_): *δ*=166.40, 165.59, 152.33, 149.29, 138.46, 131.34, 129.32, 127.21, 125.46, 124.73, 121.10, 119.76, 110.79, 110.45, 61.31, 56.18, 14.43; HRMS (ESI^+^) [*M*+H]^+^ C_18_H_20_NO_5_ calculated 330.1341 Da, found: 330.1344 *m*/*z*.

**Ethyl 3‐(2‐(3,4‐dimethoxyphenyl)acetamido)benzoate (4)**: Compound **4** was synthesized according to the general procedure (but extracted with CH_2_Cl_2_ instead of EtOAc), using 2‐(3,4‐dimethoxyphenyl)acetic acid (0.20 g, 1.02 mmol) and was obtained as a yellow solid (0.28 g, 0.83 mmol, 81 %). ^1^H NMR (500 MHz, CDCl_3_): *δ*=7.91–7.84 (m, 2H), 7.74 (d, *J*=7.7 Hz, 1H), 7.49 (d, *J*=7.2 Hz, 1H), 7.35 (t, *J*=7.8 Hz, 1H), 6.89–6.84 (m, 2H), 6.83 (s, 1H), 4.33 (q, *J*=7.1 Hz, 2H), 3.88 (s, 3H), 3.87 (s, 3H), 3.68 (s, 2H), 1.35 (t, *J*=7.1 Hz, 3H); ^13^C NMR (126 MHz, CDCl_3_): *δ*=169.80, 166.30, 149.59, 148.74, 138.00, 131.26, 129.19, 126.66, 125.48, 124.37, 121.84, 120.65, 112.59, 111.84, 61.28, 56.03, 44.43, 14.38; HRMS (ESI^+^) [*M*+H]^+^ C_19_H_22_NO_5_ calculated 344.1498 Da, found: 344.1506 *m*/*z*.

**Ethyl 3‐(2,4‐bis‐(benzyloxy)benzamido)benzoate (21)**: Compound **21** was synthesized according to the general procedure, using **16** (0.06 g, 0.18 mmol) and was obtained as a brown solid (0.02 g, 0.04 mmol, 20 %). ^1^H NMR (400 MHz, CDCl_3_): *δ*=9.92 (s, 1H), 8.30 (d, *J*=8.7 Hz, 1H), 7.94 (s, 1H), 7.71 (d, *J*=7.7 Hz, 1H), 7.61–7.21 (m, 12H), 6.77 (d, *J*=9.0 Hz, 1H), 6.73 (s, 1H), 5.19 (s, 2H), 5.14 (s, 2H), 4.37 (q, *J*=7.1 Hz, 2H), 1.39 (t, *J*=7.1 Hz, 3H).

**Ethyl 3‐(3,4‐bis‐(benzyloxy)benzamido)benzoate (22)**: Compound **22** was synthesized according to the general procedure using **17** (0.10 g, 0.30 mmol), but was not extracted. Instead the solvent of the reaction mixture was evaporated under reduced pressure and the residue directly purified by column chromatography. The desired product was obtained as a yellow solid (0.14 g, 0.28 mmol, 93 %). ^1^H NMR (400 MHz, CDCl_3_): *δ*=8.09 (s, 1H), 8.03 (d, *J*=8.1 Hz, 1H), 7.99 (s, 1H), 7.80 (d, *J*=7.7 Hz, 1H), 7.55 (d, *J*=2.0 Hz, 1H), 7.48–7.27 (m, 12H), 6.93 (d, *J*=8.4 Hz, 1H), 5.21 (s, 2H), 5.19 (s, 2H), 4.36 (q, *J*=7.1 Hz, 2H), 1.38 (t, *J*=7.1 Hz, 3H).

**Ethyl 3‐(3,5‐bis‐(benzyloxy)benzamido)benzoate (23)**: Compound **23** was synthesized according to the general procedure (but extracted with CH_2_Cl_2_ instead of EtOAc), using **18** (0.17 g, 0.50 mmol) and was obtained as a white solid (0.09 g, 0.02 mmol, 36 %). *m/z* (ESI^+^) [*M*+H]^+^ C_30_H_28_NO_5_ calculated 482.2 Da, found: 482.8 *m*/*z*.

**General procedure for the preparation of compounds 1,3, and 6 via O‐benzyl‐deprotection**: To a mixture of the respective O‐benzyl‐protected compounds in THF/MeOH (1 : 1 *v*/*v*, 30 mL mmol^−1^) was added palladium on active carbon (20 w%). The resulting suspension was stirred at room temperature for 8 h under H_2_ atmosphere (1 bar) and then filtered over Celite. The solvent was evaporated under reduced pressure and the resulting crude product purified by column chromatography on SiO_2_ (hexane/EtOAc as eluent) to afford the desired compounds **1**, **3** and **6**.

**Ethyl 3‐(2,4‐dihydroxy‐benzamido)benzoate (1)**: Compound **1** was synthesized according to the general procedure using **21** (0.05 g, 0.10 mmol) and was obtained as a grey solid (0.03 g, 0.10 mmol, 99 %). ^1^H NMR (400 MHz, [D_6_]DMSO): *δ*=12.17 (br s, 1H), 10.35 (s, 1H), 10.25 (br s, 1H), 8.33 (t, *J*=1.9 Hz, 1H), 7.95 (ddd, *J*=8.1, 2.1, 0.9 Hz, 1H), 7.90 (d, *J*=8.8 Hz, 1H), 7.70 (dt, *J*=7.8, 1.2 Hz, 1H), 7.50 (t, *J*=7.9 Hz, 1H), 6.38 (dd, *J*=8.7, 2.3 Hz, 1H), 6.33 (d, *J*=2.3 Hz, 1H), 4.33 (q, *J*=7.1 Hz, 2H), 1.33 (t, *J*=7.1 Hz, 3H). HRMS (ESI^+^) [*M*+H]^+^ C_16_H_16_NO_5_ calculated 302.1028 Da, found: 302.1027 *m*/*z*.

**Ethyl 3‐(3,4‐dihydroxy‐benzamido)benzoate (3)**: Compound **3** was synthesized according to the general procedure using **22** (0.06 g, 0.13 mmol) and was obtained as a white solid (0.03 g, 0.11 mmol, 81 %). ^1^H NMR (500 MHz, [D_6_]DMSO): *δ*=10.15 (s, 1H), 9.63 (br s, 1H), 9.27 (br s, 1H), 8.42 (s, 1H), 8.04 (d, *J*=8.0 Hz, 1H), 7.65 (d, *J*=7.7 Hz, 1H), 7.46 (t, *J*=7.9 Hz, 1H), 7.41 (s, 1H), 7.38 (dd, *J*=8.2, 1.9 Hz, 1H), 6.83 (d, *J*=8.2 Hz, 1H), 4.32 (q, *J*=7.1 Hz, 2H), 1.33 (t, *J*=7.1 Hz, 3H); ^13^C NMR (126 MHz, [D_6_]DMSO): *δ*=165.72, 165.44, 149.09, 145.01, 139.92, 130.24, 128.93, 125.51, 124.60, 123.79, 120.68, 119.77, 115.48, 114.92, 60.77, 14.21; HRMS (ESI^+^) [*M*+H]^+^ C_16_H_16_NO_5_ calculated 302.1028 Da, found: 302.1033 *m*/*z*.

**Ethyl 3‐(3,5‐dihydroxy‐benzamido)benzoate (6)**: Compound **6** was synthesized according to the general procedure using **23** (0.09 g, 0.18 mmol) and was obtained as a grey solid (0.05 g, 0.15 mmol, 86 %). ^1^H NMR (500 MHz, [D_6_]DMSO): *δ*=10.29 (s, 1H), 9.57 (s, 2H), 8.44 (t, *J*=1.9 Hz, 1H), 8.04 (ddd, *J*=8.2, 2.2, 1.0 Hz, 1H), 7.67 (dt, *J*=7.7, 1.3 Hz, 1H), 7.47 (t, *J*=8.0 Hz, 1H), 6.80 (d, *J*=2.2 Hz, 2H), 6.43 (t, *J*=2.2 Hz, 1H), 4.33 (q, *J*=7.1 Hz, 2H), 1.33 (t, *J*=7.1 Hz, 3H). ^13^C NMR (126 MHz, [D_6_]DMSO): *δ*=165.93, 165.65, 158.36, 139.64, 136.72, 130.27, 128.94, 124.62, 124.05, 120.72, 105.86, 105.62, 60.75, 14.18; Anal. calcd for C_16_H_15_NO_5_ (301.3): C, 63.78; H, 5.02; N, 4.65; found: C, 63.83; H, 5.02; N, 4.66 %; HRMS (ESI^+^) [*M*+H]^+^ C_16_H_16_NO_5_ calculated 302.1028 Da, found: 302.1033 *m*/*z*.

**Preparation of 1‐(2,4‐dihydroxyphenyl)‐2‐(3,4‐dimethoxyphenyl)ethan‐1‐one (5)**: To a solution of 2‐(3,4‐dimethoxyphenyl)acetic acid (1.57 g, 8.00 mmol, 1 equiv) in boron trifluoride diethyl etherate (15.84 mL, 56.00 mmol, 7 equiv) at 0 °C resorcinol (1.32 g, 12 mmol, 1.5 equiv) was added. The mixture was heated for 5 h at 110 °C and then cooled to 0 °C. Cold water (200 mL) was added, the resulting precipitate collected by filtration, washed with water and recrystallized from EtOH to afford **5** as a white solid (0.44 g, 1.52 mmol, 19 %). ^1^H NMR (400 MHz, [D_6_]DMSO): *δ*=12.57 (s, 1H), 10.68 (s, 1H), 7.95 (d, *J*=8.9 Hz, 1H), 6.90 (d, *J*=1.9 Hz, 1H), 6.87 (d, *J*=8.2 Hz, 1H), 6.79 (dd, *J*=8.2, 2.0 Hz, 1H), 6.39 (dd, *J*=8.8, 2.4 Hz, 1H), 6.25 (d, *J*=2.4 Hz, 1H), 4.19 (s, 2H), 3.72 (s, 3H), 3.71 (s, 3H); ^13^C NMR (101 MHz, [D_6_]DMSO): *δ*=202.41, 164.95, 164.69, 148.60, 147.63, 133.61, 127.46, 121.53, 113.36, 112.15, 111.85, 108.26, 102.49, 55.50, 55.47, 43.69; HRMS (ESI^+^) [*M*+H]^+^ C_16_H_17_O_5_ calculated 289.1076 Da, found: 289.1074 *m*/*z*.

### Biological validation

**Cell culture**: Human embryonic kidney (HEK)‐Blue hTLR2 cells (InvivoGen, Touluse, France) were used from passage 5 to 20. Stimulation of the cells with TLR2 agonists leads to production and secretion of NF‐κB‐inducible embryonic alkaline phosphatase (SEAP). The enzyme transforms the substrate QUANTI‐Blue (InvivoGen) into a blue dye that was detected by optical density (OD) at 640 nm as previously described.11a, 12 THP‐1 cells (DSMZ, Braunschweig, Germany) from passage 10 to 15 were cultured as previously reported.^13^ For the generation of THP‐1 macrophages, THP‐1 monocytes were first incubated with 25 ng/mL Phorbol 12‐myristate 13‐acetate (PMA, Sigma‐Aldrich) for 48 h and afterwards rested for 24 h. The cell lines were cultured at 37 °C in a humidified atmosphere of 5 % CO_2_ and 95 % air and were regularly tested negative for mycoplasma contamination (VenorGeM Classic Mycoplasma PCR detection kit, Minerva Biolabs, Berlin, Germany).

**TLR2 ligands**: The synthetic lipopeptides Pam_3_CSK_4_ and Pam_2_CSK_4_, lipopolysaccharide from *Escherichia coli* O111:B4 (LPS‐EB), flagellin from *Salmonella typhimurium* (FLA‐ST), the thiazoloquinoline compound CL075 and class B CpG oligonucleotide ODN2006 were purchased from Invivogen and the TLR2 antagonist CU‐CPT22 was obtained from Sigma‐Aldrich.

**Cell stimulation**: HEK‐Blue hTLR2 cells (4×10^4^ cells/well) and THP‐1 macrophages (2×10^5^ cells/well) were cultured in 96‐well plates and 24‐well plates (TPP, Trasadingen, Switzerland). After cells were washed with phosphate‐buffered saline (PBS, Sigma‐Aldrich), cell stimulation was done in OptiMEM (ThermoFisher Scientific). The tested compounds and CU‐CPT22 (50 mM) were dissolved in DMSO. Final DMSO concentrations in cell culture were below 0.2 % (*v*/*v*). Vehicle controls showed no significant difference to nonstimulated controls (data not shown). For co‐stimulation of TLR2 agonists and tested modulators, cells were preincubated with the compounds or CU‐CPT22 for 1 h and afterwards stimulated with the agonists.

**ELISA**: Commercially available ELISA kits were used for detecting human IL‐8 levels in cell culture supernatants (ELISA‐Ready Set Go, Invitrogen by Thermo Fisher Scientific).

**Cell viability**: Cell viability was assessed by the MTT assay in HEK Blue hTLR2 cells as previously described.^14^ 10 % (*v*/*v*) DMSO (Carl Roth) served as control and the viability of untreated cells was defined as 100 %.

**Statistical analysis**: Data of the bar charts are shown as mean+SD. Potency (IC_50_) data are presented as mean with the confidence interval (95 %). Statistical analysis was done by using GraphPad Prism 6.0 (GraphPad software, San Diego, USA). Nonlinear regression was used to plot and analyze concentration‐response curves and to obtain IC_50_ values.

**Computational methods**: The crystal structure of the heterodimer of TLR2‐TLR1 with bound Pam_3_CSK_4_ (PDB ID: 2Z7X)^15^ was retrieved from the Protein Data Bank^16^ and used for docking studies with the tested compounds. Prior to docking the TLR1 monomer, all ligands and water molecules were removed using *Molecular Operating Environment* (MOE2019, Chemical Computing Group, Montreal, QC, Canada). The TLR2 monomer was protonated using the “Protonate 3D” application included in MOE2019. The GOLD Suite v.5.2 (Cambridge Crystallographic Data Centre, Cambridge, UK)^17^ was used for docking with the GoldScore^18^ as scoring function with “slow” parameters. Binding poses were minimized (MMFF94 force field)^19^ and further analyzed in LigandScout 4.2 (Inte:ligand, Vienna, Austria).^20^

## Conflict of interest

The authors declare no conflict of interest.

## Supporting information

As a service to our authors and readers, this journal provides supporting information supplied by the authors. Such materials are peer reviewed and may be re‐organized for online delivery, but are not copy‐edited or typeset. Technical support issues arising from supporting information (other than missing files) should be addressed to the authors.

Supplementary
