# Supplementary material for: Biological Characterization, Mechanistic Investigation and Structure‐Activity Relationships of Chemically Stable TLR2 Antagonists
Source: ChemMedChem. 2020 Jun 3;15(14):1364–71. doi: 10.1002/cmdc.202000060 (PMC7496872; doi:10.1002/cmdc.202000060)
Supplement: Supplementary file 1 — Supplementary [file CMDC-15-1364-s001.pdf]

ChemMedChem

Supporting Information

Biological Characterization, Mechanistic Investigation and Structure-Activity Relationships of Chemically Stable TLR2 Antagonists

Marcel Bermudez[+], Maria Grabowski[+], Manuela S. Murgueitio, Markus Tiemann, Péter Varga, Thomas Rudolf, Gerhard Wolber,* Günther Weindl,* and Jörg Rademann*© 2020 The Authors. Published by Wiley-VCH Verlag GmbH & Co. KGaA.
This is an open access article under the terms of the Creative Commons Attribution License, which permits use, distribution and reproduction in any medium, provided the original work is properly cited.

Supporting Information

Contents.

1 Supplementary Figures 2
1.1 Supplementary Figure 1: Agonistic activity and cell viability 2
1.2 Supplementary Figures 2-5: Docking poses of analyzed compounds 3

1 Supplementary Figures

1.1 Supplementary Figure 1: Agonistic activity and cell viability

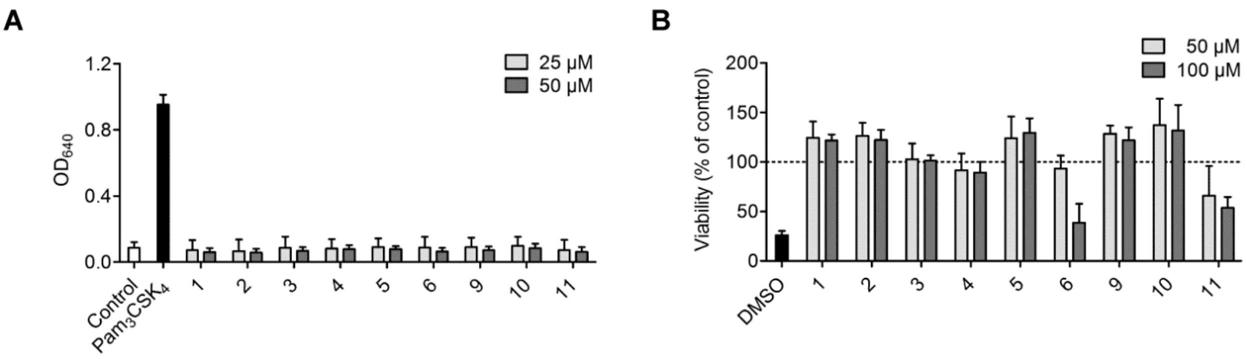

Supplementary Figure 1. Compound **6** shows no agonistic activity at TLR2 and does not affect cell viability up to 50 µM. (A) HEK-Blue hTLR2 cells were stimulated with Pam_3CSK_4 (10 ng/ml) or the compounds **1**-**9** for 24 h. SEAP production was detected by QUANTI-Blue as OD at 640 nm. (B) HEK-Blue hTLR2 cells were stimulated with DMSO (10% v/v) or the compounds for 24 h. Cell viability was determined by MTT assay and normalized to unstimulated cells (dashed line). Mean +SD (n=3).

1.2 Supplementary Figures 2-5: Docking poses of analyzed compounds

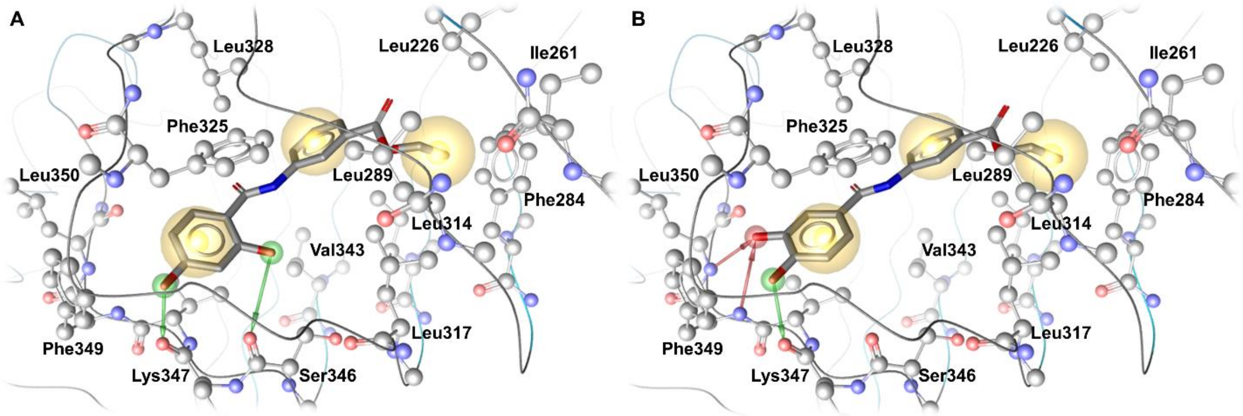

Supplementary Figure 2. Predicted binding pose of compounds **1** (A) and **3** (B). Protein residues are depicted in ball and stick mode, the compound as sticks. Protein-ligand interactions are color- and shape-coded (yellow sphere – hydrophobic contact area, green arrow – H-bond donor, red arrow – H-bond acceptor).

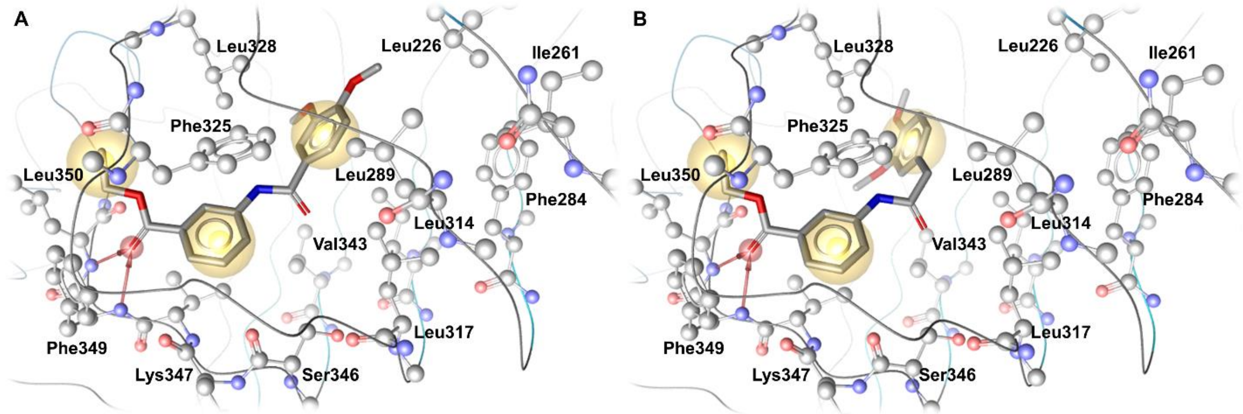

Supplementary Figure 3. Flipped binding mode of compounds **2** (A) and **4** (B). Protein residues are depicted in ball and stick mode, the compound as sticks. Protein-ligand interactions are color- and shape-coded (yellow sphere – hydrophobic contact area, green arrow – H-bond donor, red arrow – H-bond acceptor).

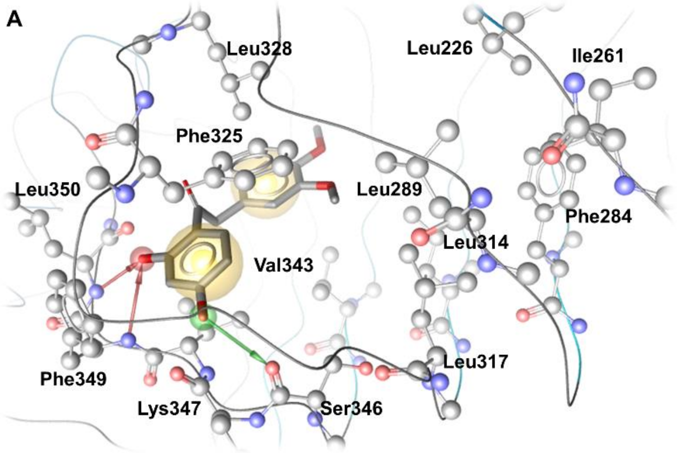

Supplementary Figure 4. Predicted binding mode of compound **5**. Protein residues are depicted in ball and stick mode, the compound as sticks. Protein-ligand interactions are color- and shape-coded (yellow sphere – hydrophobic contact area, green arrow – H-bond donor, red arrow – H-bond acceptor).

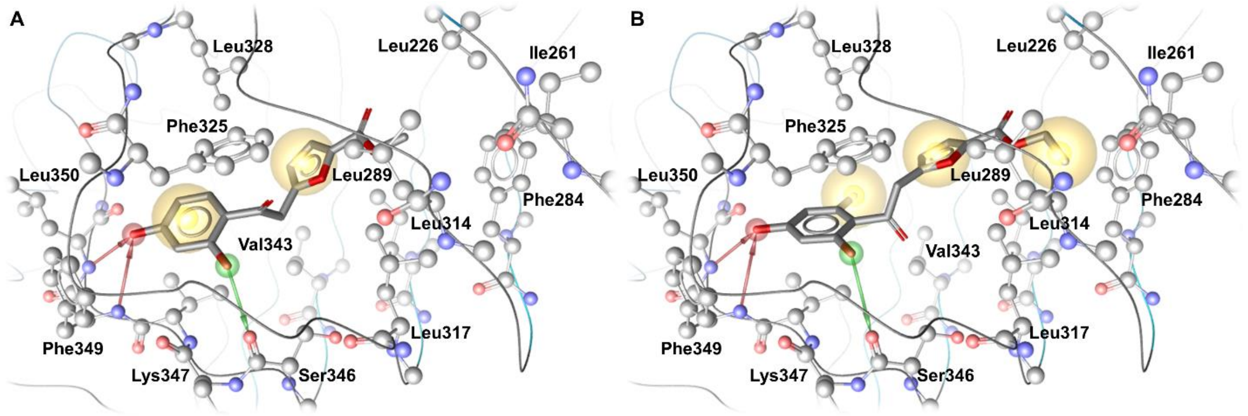

Supplementary Figure 5. Binding mode of compounds **7** (A) and **8** (B). Protein residues are depicted in ball and stick mode, the compound as sticks. Protein-ligand interactions are color- and shape-coded (yellow sphere – hydrophobic contact area, green arrow – H-bond donor, red arrow – H-bond acceptor).
